# Feasibility of Using Research Electronic Data Capture (REDCap) to Collect Daily Experiences of Parent-Child Dyads: Ecological Momentary Assessment Study

**DOI:** 10.2196/42916

**Published:** 2023-11-09

**Authors:** Yola El Dahr, Florence Perquier, Madison Moloney, Guyyunge Woo, Roksana Dobrin-De Grace, Daniela Carvalho, Nicole Addario, Emily E Cameron, Leslie E Roos, Peter Szatmari, Madison Aitken

**Affiliations:** 1 Cundill Centre for Child and Youth Depression Centre for Addiction and Mental Health Toronto, ON Canada; 2 Faculty of Arts & Science University of Toronto Toronto, ON Canada; 3 Department of Psychology Toronto Metropolitan University Toronto, ON Canada; 4 Schulich School of Medicine & Dentistry Western University London, ON Canada; 5 Department of Psychology University of Manitoba Winnipeg, MB Canada; 6 Children’s Hospital Institute of Manitoba Winnipeg, MB Canada; 7 Department of Psychiatry University of Toronto Toronto, ON Canada; 8 Department of Psychology York University Toronto, ON Canada

**Keywords:** ambulatory assessment, children, ecological momentary assessment, longitudinal, parents, survey

## Abstract

**Background:**

Intensive longitudinal data collection, including ecological momentary assessment (EMA), has the potential to reduce recall biases, collect more ecologically valid data, and increase our understanding of dynamic associations between variables. EMA is typically administered using an application that is downloaded on participants’ devices, which presents cost and privacy concerns that may limit its use. Research Electronic Data Capture (REDCap), a web-based survey application freely available to nonprofit organizations, may allow researchers to overcome these barriers; however, at present, little guidance is available to researchers regarding the setup of EMA in REDCap, especially for those who are new to using REDCap or lack advanced programming expertise.

**Objective:**

We provide an example of a simplified EMA setup in REDCap. This study aims to demonstrate the feasibility of this approach. We provide information on survey completion and user behavior in a sample of parents and children recruited across Canada.

**Methods:**

We recruited 66 parents and their children (aged 9-13 years old) from an existing longitudinal cohort study to participate in a study on risk and protective factors for children’s mental health. Parents received survey prompts (morning and evening) by email or SMS text message for 14 days, twice daily. Each survey prompt contained 2 sections, one for parents and one for children to complete.

**Results:**

The completion rates were good (mean 82%, SD 8%) and significantly higher on weekdays than weekends and in dyads with girls than dyads with boys. Children were available to respond to their own survey questions most of the time (in 1134/1498, 75.7% of surveys submitted). The number of assessments submitted was significantly higher, and response times were significantly faster among participants who selected SMS text message survey notifications compared to email survey notifications. The average response time was 47.0 minutes after the initial survey notification, and the use of reminder messages increased survey completion.

**Conclusions:**

Our results support the feasibility of using REDCap for EMA studies with parents and children. REDCap also has features that can accommodate EMA studies by recruiting participants across multiple time zones and providing different survey delivery methods. Offering the option of SMS text message survey notifications and reminders may be an important way to increase completion rates and the timeliness of responses. REDCap is a potentially useful tool for researchers wishing to implement EMA in settings in which cost or privacy are current barriers. Researchers should weigh these benefits with the potential limitations of REDCap and this design, including staff time to set up, monitor, and clean the data outputs of the project.

## Introduction

### Overview

Researchers are increasingly using intensive longitudinal data, in which responses are collected frequently across an interval of days or weeks, to understand health-related risk and protective factors [1]. One method commonly used to collect intensive longitudinal data is ecological momentary assessment (EMA) [1]. EMA is a collection of research methodologies that take multiple assessments from participants in their natural environment and in their current state [2,3].

EMA has 3 main advantages over traditional survey designs. First, the use of frequent measurements in participants’ natural environments reduces recall, rater biases, and memory errors [2,4-6]. For example, EMA ratings correlate higher than retrospective recall ratings with accelerometer measures of physical activity [7-9]. Similarly, information collected through EMA ratings predicts children’s response to treatment for depression or anxiety above and beyond traditional self-report symptom measures [10]. Second, ratings collected through EMA are more ecologically valid than traditional surveys since they can include information on contextual factors that may influence ratings [11,12]. For example, a review of EMA use in the study of suicidal thoughts and behavior identified sleep quality and negative affect as short-term predictors of suicidal behavior [13]. In mental health research, EMA studies can therefore provide a more nuanced understanding of the presentation in question, which may not be accurately represented in questionnaires administered within a research environment. Third, data collected through EMA can be used to identify dynamic associations between variables over time [1-3], including potential causal associations [14].

Despite its usefulness, current barriers make the use of EMA challenging. One potential barrier is the cost associated with the programs and applications that enable EMA data collection. EMA is often carried out by installing an application on participants’ smartphones [15]. Some of the current, most versatile software programs available in the market cost between US $1000 to $6000 [16], which may be prohibitive for many research teams. In addition, EMA applications may present privacy concerns [17-19]. For example, EMA applications may store participant data on external servers [15], including servers outside the country in which the researchers are working. Data are subject to the privacy laws of the country in which they are stored, which may vary in their level of protection and requirements for disclosure [20]. Storing data internally on a smartphone also poses a security risk as devices may be lost, misplaced, or stolen [19]. Another privacy concern is that EMA applications may collect extraneous data, such as phone use or location, when installed on participants’ smartphones [17]. Addressing cost and privacy barriers may increase the use of EMA, leading to results that support a more nuanced understanding of different health behaviors.

### Research Electronic Data Capture

The use of existing and accessible software may help to overcome both cost and privacy barriers to using EMA. One such example is Research Electronic Data Capture (REDCap), developed by Vanderbilt University to improve the ease of capturing data for clinical research projects [21]. This web-client platform can be accessed through any device with a secure internet connection, and the easy-to-use tools require minimal programming abilities [22]. REDCap licenses are issued for free to nonprofit organizations that join the REDCap consortium. It is important to note that there are costs associated with running a REDCap server at an institution, which include costs for personnel, hardware, and network connections, among others.

Features of REDCap also enhance privacy and data security. REDCap is compliant with the Health Insurance Portability and Accountability Act and has features that can safeguard personal health information [23]. These include the ability to limit access to identifying or sensitive information to specific team members and a 2-step authentication process. When building surveys within REDCap, researchers can mark specific fields as identifiable, allowing them to export only deidentified data. REDCap also stores data on servers designated by the institution for which it is set up, allowing researchers to ensure that data are protected by their country’s own privacy laws.

An additional benefit of using REDCap is that it is a flexible and self-sufficient platform [24]. REDCap has a variety of features, such as surveys that include branching logic, the ability to design a matrix of questions for longer surveys, and the action of capturing electronic signatures for documenting informed consent, if required by institutional review boards [21]. Furthermore, REDCap has tools that allow users to easily export data to another statistical platform, such as Excel (Microsoft Corp), SPSS (IBM Corp), or R (The R Foundation). Integration with third-party applications also increases REDCap’s capabilities. Especially relevant for EMA, REDCap can interface with Twilio, a third-party application, to automatically send survey notifications to participants’ mobile phones through SMS text message [16] for a small fee per message (at the time of writing, approximately US $0.0079 per text sent). Participants are automatically sent an SMS text message containing a link to the survey at time intervals that are determined by the research team.

While REDCap is a useful tool for data collection, researchers might experience challenges in trying to accommodate a longitudinal EMA design using its built-in features when working with older versions of the program [16]. As REDCap was initially developed for clinical trials, there are no easy-to-implement methods for sending out survey prompts frequently over a short period of time in these older versions. In addition, EMA studies are often carried out in a remote fashion, given that they require capturing data from participants in real time. Since this reduces the need for routine interactions with participants, it presents an opportunity for researchers to expand the reach of their recruitment, potentially spanning multiple time zones. Lastly, some participants may prefer to receive survey notifications through email, whereas others may prefer delivery by SMS text message. Setting up 2 separate delivery methods creates additional programming challenges that the REDCap administrator must navigate.

Ultimately, REDCap provides an extensive tool kit of features that can be overwhelming to navigate for new or inexperienced users. In order to increase the capacity among health and social science researchers to use intensive longitudinal methods, it is important to share simplified designs that use recognizable features.

### This Study

Despite the many potential benefits of ambulatory assessment methods such as EMA, the cost of software to facilitate EMA data collection and associated privacy concerns remain barriers to its widespread use. REDCap is a flexible tool that may allow researchers to overcome some of these key barriers to EMA implementation. Aside from 1 existing study that did not require survey delivery across time zones and used only a single survey per day [16], there is limited guidance available regarding how to use REDCap for EMA. The present report provides an overview of how REDCap can be used for EMA in a fully remote–capable, parent- and child-report EMA study. We present detailed instructions for survey setup, along with evidence of feasibility and information on user behavior based on a vanguard sample of 66 participants in an ongoing parent and child EMA study.

## Methods

### Recruitment

Participants were children aged between 9 and 13 years and their parents, who were part of a larger study of factors associated with variation in children’s emotional-behavioral functioning during the COVID-19 pandemic. Participants were recruited from the Canadian Healthy Infant Longitudinal Development (CHILD) cohort study [25], a multicenter, prospective, longitudinal birth cohort study that follows participants from pregnancy to the present day in 4 Canadian centers. CHILD was designed to understand the development of chronic disease by collecting data from various questionnaires, assessments, and biological samples [25,26]; however, it has since been used to look at health and development more broadly, including factors that influence children’s mental health.

The CHILD participants (n=3542) from which the present sample was taken are predominantly White (2532/3542, 71.5%), with parents having at least a college or university-level education (5997/6794, 88.3%) [25]. Participants were recruited from CHILD if (1) the parent consented to be contacted about future studies; (2) the age 8 CHILD data collection visit was completed before January 2020; and (3) they were residing in Canada.

At the time of data analysis, recruitment had only been conducted at 2 out of the 4 CHILD sites: Edmonton, Alberta, and Vancouver, British Columbia. Recruitment did not begin simultaneously at all sites due to differences across sites in the timing of securing agreements and ethical approvals. A total of 619 participants from the 2 initial sites were emailed a brief communication about this study, including a REDCap link to our e-consent form. Participants reported that they were in either the Mountain Standard Time or Pacific Standard Time zones. The current sample includes 66 parent-child dyads who had completed the study at the time of writing.

**Figure 1:**
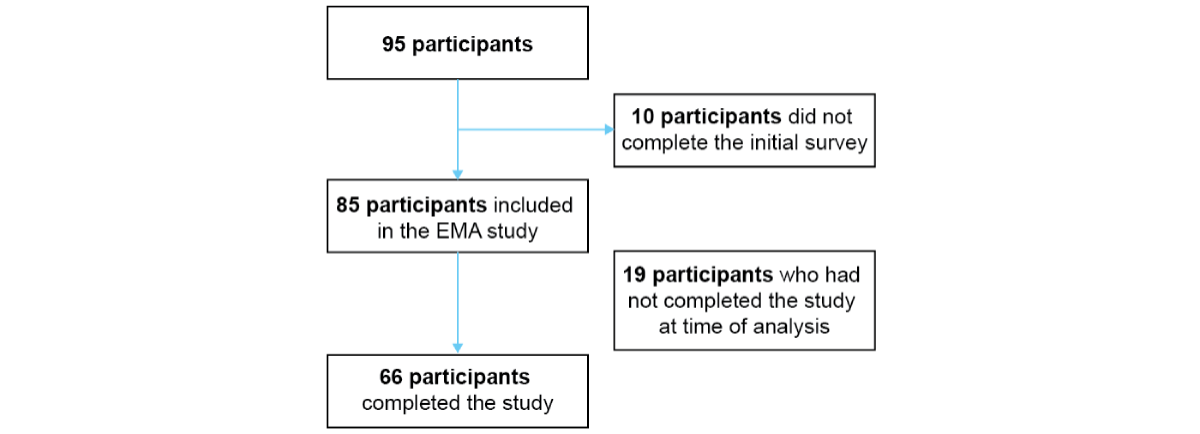
Flowchart of inclusion and exclusion of participants in the current analysis. EMA: ecological momentary assessment.

For all participants, EMA data were collected over a 2-week period after the initial questionnaire was completed. The present analysis was carried out on July 4, 2022, and included all participants who completed the study between May 31 and June 16. More than 89% (85/95) of eligible participants completed the initial questionnaire and agreed to participate. Among those, 78% (66/85) of participants had completed the whole survey at the time of analysis (Figure 1).

### Measures

#### Initial Survey

Parents provided basic demographic information, along with their time zone, relationship to the child, and other study-specific information (eg, COVID-19 vaccination status). They also completed the 6-item Kessler Screening Questionnaire [27], a measure of psychological distress, with each item rated on a 5-point Likert scale. Parents indicated whether they would like to receive the links to the EMA surveys through email or SMS text message.

#### EMA Surveys

Parents were asked to estimate the amount of time their child spent in various activities (doing things with the parent, interacting face-to-face with family members who live outside the home, in meaningful face-to-face interactions with peers, in web-based or e-learning, watching television or digital media, and sleeping), and to rate their own stress levels, as well as their child’s positive and negative emotions and disruptive behavior, all over the previous 12 hours. Children were asked to rate how much time they spent playing or talking with friends face-to-face, their positive and negative emotions, and their interactions with their participating parent (warmth, harsh parenting, and inconsistent parenting) over the past 12 hours on a 5-point scale ranging from “not at all” to “a lot.” EMA items were adapted from the Positive and Negative Affect Schedule for Children [28], the Coronavirus Health Impact Survey [29], and from existing, single-item measures of relevant constructs [30-37]. Based on pilot testing within our research team, EMA surveys took approximately 5 minutes each to complete by both parent and child.

### Procedure

#### Overview

After completing the initial survey, parents received survey notifications beginning the following day through their preferred delivery method (email or SMS text message), twice daily (at 7:30 AM and 7:30 PM local time) for 14 days. The timings of the surveys were selected based on input from parent advisors to accommodate the availability of as many parent-child dyads as possible, since most children would be traveling to school in the mornings. The evening time was then selected to be spaced 12 hours from the morning survey.

At the beginning of each survey, parents were asked if their child had been staying outside the home for the past 12 hours; if this was the case, the survey ended. After completing the parent items, parents were asked if their child was present and willing to answer survey questions; if so, children then completed EMA ratings using the same device. Parents who had not responded received 2 reminders at 30-minute intervals. The survey remained open for 3 hours following the initial notification to allow parents enough time to provide their responses and to accommodate potential differences in family schedules. We provide an overview of our REDCap setup in Multimedia Appendix 1. These instructions are based on REDCap version 11.1.21.

#### Survey Setup in the REDCap Online Designer

Since this study recruited participants from across Canada, the REDCap setup had to accommodate individuals from 6 time zones. Surveys for different time zones must be set up separately to facilitate the programming of survey notifications later on. We set up a morning survey and an evening survey for each of the time zones, leaving us with 6 morning surveys and 6 evening surveys in total. To differentiate between surveys for different time zones, we included the time zone abbreviation in each of our survey titles.

#### Settings to Consider in Project Setup and the Online Designer

Using the Project Setup page, we enabled both Surveys and Longitudinal Data Collection for this project. We then identified an email field (from the initial survey) in the “Enable optional modules and customizations” section to email participants the survey links should they choose this option. Then, in the Online Designer, we enabled all our data collection instruments as surveys. In each of the morning and evening surveys, we added a “Begin new section” field that separated the questions for parents from those of the children. Then, we accessed the Survey Settings of all the morning and evening surveys to edit some functions. First, we selected the “Enhanced radios and checkboxes” option under the Survey Design Options section. This enables larger buttons in the survey to appear, which enhances the user experience for participants completing their surveys on a mobile device. Second, under Survey Customizations, we selected “Multiple pages (display 1 section per page)” so that questions for child participants appear on their own page. Finally, under the Survey Access section, we imposed a 3-hour time limit for survey completion (from the time of survey delivery) using the “Time limit for survey completion” option.

#### Twilio Setup

Twilio was used to send surveys to participants who selected notifications through SMS text message. From the Project Setup page, we accessed the Twilio main menu to configure its settings. We enabled surveys to be sent as web pages only. We also identified which field asks for participants’ preferred method of survey delivery (email or SMS text message), as well as the field in which participants’ cell phone numbers could be found (from the initial survey).

#### Events and Instruments Setup

We split the project into 6 arms, each representing a time zone in Canada. We had 29 events in each of the arms. The first event we defined was the baseline event, and all subsequent events were EMA morning and evening surveys for days 1-14. In each arm, the Initial Questionnaire and the Parent Information and Contact Preferences form were assigned to the baseline event. We then designated the appropriate morning and evening EMA surveys for each of the arms.

#### Setup of Automated Survey Notifications

In the Online Designer, we used the Automated Invitations feature to set up the survey notifications for each of the events we defined in the previous step. We enabled up to 2 reminders for each of the surveys, programmed to be sent 30 minutes and 1 hour after the initial survey notification was delivered if the participant had not responded. Further details are provided in Multimedia Appendix 1.

#### Staff Time

Research staff members were involved in the setting up, piloting, and monitoring phases of the study. Setup in REDCap required a few days of work from building the instruments to programming the notifications, to then performing error checking on the project. Piloting lasted a total of 2 weeks, during which staff members were assigned a time zone to sign up for and pilot its surveys, delivered through email or SMS text message. In this phase, we ensured that survey delivery was successful at the desired times and that surveys were suitable for completion on both laptops and mobile phones. Once piloting was complete, we began the data collection phase. This required 1 staff member to monitor new sign-ups daily through REDCap and check for any errors that were flagged by the system. Daily monitoring did not take more than a few minutes each time. Finally, data clean up took a total of 2 days.

### Statistical Analysis

The database was exported to Stata (StataCorp) using the automated export procedure. Adherence to the study was measured in terms of the number of EMA responses available across the 14 days (up to a maximum of 28), completion rates at each time point, and child availability to fill out their part of the questionnaire. Differences in completion rates according to time of day (morning or evening), weekends and weekdays, and notification type (SMS text message or email) were identified using univariate chi-square tests for categorical variables and the nonparametric Wilcoxon Mann-Whitney test for numeric variables. We also examined user behavior, including average response time (computed as the time lag between the first notification and the response time logged in REDCap). Finally, we compared included and excluded participants’ characteristics according to gender, income, and type of notification (email or SMS text message). For categorical variables, we used Fisher exact test of independence to test for differences, as some expected numbers were less than 5.

### Ethical Considerations

This study was approved by the last author’s (MA) (Centre for Addiction and Mental Health) research ethics board (003-2022) and the ethics boards at each of the respective CHILD study sites (University of Alberta, Pro00117899_AME1; UBC Children's & Women’s Research Ethics Board, H07-03120). All participants participated voluntarily. Parents provided their consent, and children provided their assent using our hospital’s e-consent framework in REDCap. Identifiers, such as email and phone number, were collected initially and stored in REDCap to facilitate survey delivery. Fields that collected these data were marked as identifiers in the REDCap Online Designer to filter them out from any data exports and ensure that the main database is deidentified. Participating families were paid up to CAD $90 (US $67) in the form of e-gift cards at the end of this study, depending on the number of surveys completed (CAD $10 [US $7.40] for completing the baseline questionnaire, CAD $2.50 [US $1.85] for every survey completed, and an extra CAD $10 [US $7.40] for completing 21 or more out of the 28 daily surveys).

## Results

### Surveys Available

From May 31 to July 4, 2022, a total of 1564 records were received from 66 parent-child dyads (66 baseline and 1498 EMA records). EMA records split almost equally between morning (n=739, 49.3%) and evening surveys (n=759, 50.7%). On 108 (7.2%) occasions, the child was staying outside the home for the previous 12 hours, and no EMA ratings were collected.

### Participant Characteristics

Most parent participants were women (n=64, 97%) and all parents were biologically related to the child. Children ranged in age from 9 to 13 years old. The sample was of relatively high socioeconomic status, with 82% (54/66) having a total annual family income of CAD $90,000 (US $66,628) or more. The majority of participants spoke English at home, with 1 family speaking both English and French and 1 family speaking Russian. See Table 1 for detailed sample characteristics. More than half of the participants made the choice to receive the link to the EMA surveys by SMS text messages (34/66, 52%), but the proportion of participants who preferred to be notified by email was substantial (32/66, 48%).

Compared to the 66 participants included, 19 participants who had not completed the study at the time of analysis had 18.8 (SD 8.0) records on average (vs 22.7, SD 7.7 for participants included). Participants who had not completed the study yet had a slightly higher income (4/12, 21% reported an annual income of CAD $90,000 [US $66,628]) than participants who completed it (12/66, 18%). No difference was found according to gender, or notification type. See Table 2 for detailed differences between included and excluded participants.

There were no differences in participants’ characteristics between the 2 sites. See Table 3 for detailed comparisons between participant characteristics from the 2 sites.

**Table 1:** Sociodemographic characteristics of participants (n=66). A currency exchange rate of CAD $1=US $0.74 is applicable.

Characteristics			Value, n (%)
**Parent’s gender^a^**			
	Woman	64 (97)	
	Man	1 (2)	
	Genderqueer	1 (2)	
	Gender fluid	1 (2)	
	PNTA^b^	1 (2)	
**Child’s gender^a^**			
	Girl	21 (32)	
	Boy	43 (65)	
	Nonbinary	1 (2)	
	Gender expansive	2 (3)	
**Language**			
	English only	64 (97)	
	English and French	1 (2)	
	Russian	1 (2)	
**Family income (CAD $)**			
	0-29,999	1 (2)	
	30,000-59,999	2 (3)	
	60,000-89,999	9 (14)	
	90,000-119,999	18 (27)	
	120,000-149,999	9 (14)	
	150,000 or more	27 (41)	
**Time zone**			
	Pacific Standard Time	11 (17)	
	Mountain Standard Time	55 (83)	

^a^Percentages do not add up to 100 due to rounding and the option for participants to select multiple options.

^b^PNTA: prefer not to answer.

**Table 2:** Differences in included and excluded participants (n=85). A currency exchange rate of CAD $1=US $0.74 is applicable.

Participant characteristics			Excluded (n=19)		Included (n=66)		*P* value	
**Gender, n (%)**								.14^a^
	Girl	10 (53)		21 (32)				
	Boy	8 (42)		43 (65)				
	Other	1 (5)		2 (3)				
**Income (CAD $), n (%)**								.01^a^
	<90,000	12 (63)		54 (82)				
	≥90,000	4 (21)		12 (18)				
	No answer	3 (16)		0 (0)				
Number of records, mean (SD)			18.8 (8.0)		22.7 (7.7)		.004^b^	
**Notification type, n (%)**								.80^a^
	Email	8 (42)		32 (49)				
	SMS text message	11 (58)		34 (52)				

^a^Fisher exact test.

^b^Nonparametric Wilcoxon Mann-Whitney test.

**Table 3:** Differences in participant characteristics according to site (n=66). A currency exchange rate of CAD $1=US $0.74 is applicable.

Participants’ characteristics		Vancouver (n=11)	Edmonton (n=55)	*P* value	
**Gender, n (%)**					.16^a^
	Girl	1 (9)	20 (36)		
	Boy	10 (91)	33 (60)		
	Other	0 (0)	2 (4)		
**Income (CAD $), n (%)**					>.99^a^
	<90,000	9 (82)	45 (82)		
	≥90,000	2 (18)	10 (18)		
Number of records, mean (SD)		20.2 (11.0)	23.2 (6.9)	.80^b^	
**Notification type, n (%)**					>.99^a^
	Email	5 (46)	27 (49)		
	SMS text message	6 (55)	28 (51)		

^a^Fisher exact test.

^b^Nonparametric Wilcoxon Mann-Whitney test.

### Completion Rates

Participants completed on average 23 (SD 8) of the 28 EMA assessments, and more than half of the sample (37/66, 56%) completed at least 26 assessments. The number of assessments did not differ according to total family income (mean 22.5, SD 8.4 for participants whose total family income was more than CAD $90,000 [US $66,628] vs mean 23.8, SD 3.4 for participants with lower income; *z* score=1.12; *P*=.27) but was significantly higher when the child identified as a girl compared to when the child identified as a boy (mean 24.9, SD 6.2 vs mean 21.7, SD 8.4; n=64; *z* score=2.37; *P*=.02).

**Figure 2:**
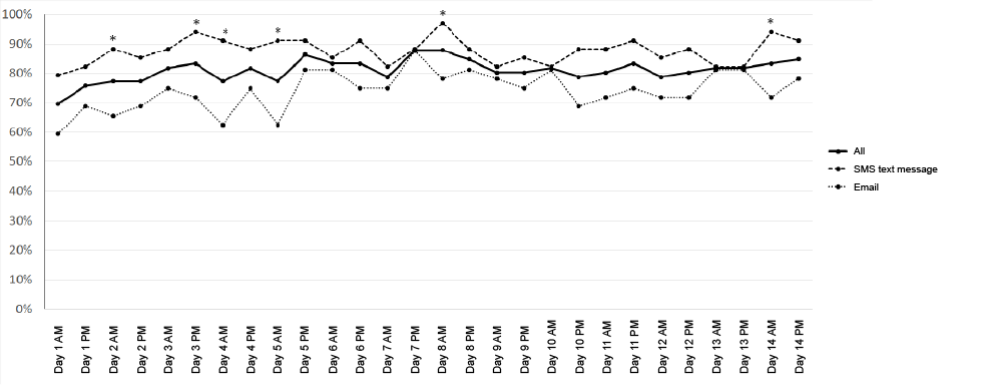
Completion rates by time point and notification type. “*” denotes a significant difference in response rates between SMS text messages and emails (*P*<.05).

Completion rates were similar across study time points, varying between 70% (46/66) and 88% (58/66) in the morning and 76% (50/66) and 88% (58/66) in the evening (Figure 2). The completion rate was higher on weekdays (Monday to Friday) than on weekend days (1087/1320, 82.4% vs 411/523, 77.8%; *χ*^2^_1_=4.9; *P*=.02). Participants notified by SMS text message had higher rates of completion at most time points, with significant differences being found on several morning occasions compared to participants notified by email. Participants notified by SMS text message also had a significantly higher number of assessments available compared to participants notified by email (mean 24.5, SD 6.5 vs mean 20.8, SD 8.5; *z* score=–2.53; *P*=.01).

Children were available to answer their own EMA survey questions in 75.7% (1134/1498) of assessments. Child availability was significantly higher in the evening than in the morning assessments (603/759, 79.5% vs 531/739, 71.9%; *χ*^2^_1_=11.7; *P*=.001). No difference in child availability was found according to notification type (512/664, 77.1% in the email vs 622/834, 74.6% in the SMS text message group; *χ*^2^_1_=1.3; *P*=.26) between weekdays and weekends (respectively, 817/1087, 75.2% and 317/411, 77.1%; *χ*^2^_1_=0.6; *P*=.43), or according to child gender (390/522, 74.7% in girls vs 720/933, 77.2% in boys; n=64; *χ*^2^_1_=1.1; *P*=.29).

### Average Response Time

The average response time was computed considering 1494 records with available time data (4 records had no logged time in REDCap, as the user was disconnected while filling out the questionnaire). The average response time was 47.0 (SD 52.3) minutes after the initial survey prompt. Response time was significantly shorter in the evening (mean 43.1, SD 40.3 minutes) than in the morning (mean 51.0, SD 62.1 minutes; *z* score=2.11; *P*=.03) and when participants were notified by SMS text message (mean 38.1, SD 41.30 minutes) than when notified by email (mean 58.2, SD 61.7 minutes; *z* score=9.33; *P*<.001).

**Figure 3:**
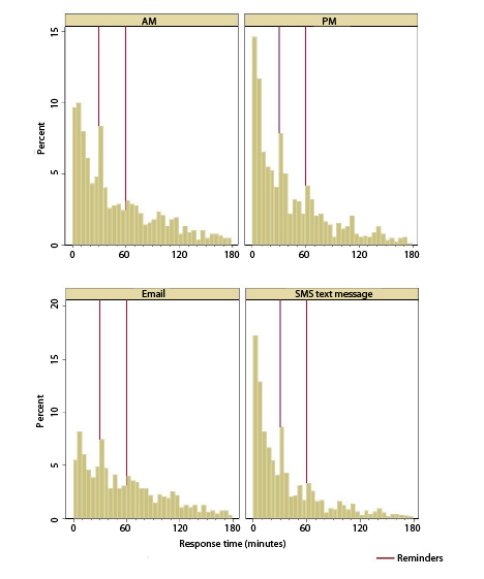
Response time by time of day and notification type. Seven records with a response time higher than 3.5 hours (due to technical problems at Day 1 AM assessment) are not presented. AM=morning time; PM=evening time.

The distribution of assessments completed according to response time (in 5-minute intervals) is presented in Figure 3. The proportion of assessments completed in the first 5 minutes was significantly higher for the evening than for the morning assessments (106/757, 14% vs 77/737, 10.5%; *χ*^2^_1_=4.4; *P*=.04), and for participants notified by SMS text message rather than email (143/831, 17.2% vs 40/663, 6%; *χ*^2^_1_=42.9; *P*<.001). Assessments completed after 2 hours represented 7.8% (116/1494) of the records and were significantly less frequent in the evening than in the morning (47/757, 6.2% vs 69/737, 9.4%; *χ*^2^_1_=5.2; *P*=.02) and when participants were notified by SMS text message rather than email (51/831, 6.1% vs 65/663, 9.8%; *χ*^2^_1_=6.9; *P*=.009). A visual inspection of Figure 3 shows that the first reminder was followed by a substantial increase in the proportion of assessments completed, whereas the second reminder appears to have enhanced completion only in the evening and in the participants notified by SMS text message. An overview of key takeaways and future research directions is presented in Textbox 1.

**Key takeaways and directions for future research.**

**Key takeaways**

- Research Electronic Data Capture (REDCap) can accommodate ecological momentary assessment (EMA) studies, including those that recruit across multiple time zones, require little to no communication with participants, and involve both parents and children.
- The use of REDCap addresses privacy and cost concerns that are often associated with EMA studies that require the use of an application.
- It is important to offer participants more than one mode of survey delivery. SMS text messages and email survey delivery were almost equally chosen in our sample. Completion rates were higher among participants who chose SMS text message delivery.
- Implementing reminders in the project design enhances survey completion rates.
- This design is most useful to users of REDCap who are new to or inexperienced with using REDCap but would like to implement an EMA study design.
- Institutions that are part of the REDCap consortium are likely to be running different versions of REDCap, some more advanced than others. It is important to communicate designs that involve easily recognizable features of REDCap that can be used regardless of the REDCap version.

**Implications for future research using EMA**

- Future studies can build on this design to develop projects that require less time to build and produce cleaner data outputs.
- Future EMA studies can investigate whether participants from rural areas have similar response rates and access to the required technology as participants from urban areas.
- Future studies can focus on why response rates in EMA studies vary by gender and suggest methods to ensure all participants are equally engaged.

## Discussion

### Overview

Ambulatory assessment methods, such as EMA, offer a number of potential benefits, such as decreased recall errors, the ability to collect more ecologically valid data in real-world settings, the collection of intensive longitudinal data that can be used to examine predictive associations over time, and improved prediction of mental health treatment outcomes [2,10,19]. We have developed a method for using a customizable data capture tool (REDCap) to collect EMA data that may allow researchers to overcome some of the cost and privacy barriers that make implementation of EMA methods challenging. By providing details of our survey setup in REDCap, we aim to increase the ability of other researchers to use similar methods in the future. This design would be most useful to researchers who are unfamiliar with or new to using REDCap, as it uses features that are easily accessible and recognizable by users. Our results also provide information on parent and child completion rates and respondent behavior.

Our preliminary results support the feasibility of using EMA with parents and children. Completion rates for both parents and children were high. Moreover, we did not observe a decline in completion rates across the 2-week study period or a difference in completion rates by family income. It is important to note that our relatively high completion rates may be in part due to offering financial compensation based on the number of surveys completed [38] or to our recruitment from a cohort of families already engaged in another research study. However, our completion rates are comparable to those reported in previous child and parent EMA studies. Heron and colleagues [39] reported an average survey completion rate of 76% among youth participants involved in 54 different EMA studies. Similarly, an EMA study that prompted parents to answer questions about their child’s affect 3 times daily for 28 days found that parents completed an average of 83% of the 84 possible assessments [40]. Our sample also had a higher than average socioeconomic status compared to the Canadian population and was largely urban and suburban [41]. These participants likely had ready access to mobile devices with internet access and may have faced fewer challenges to study participation than participants from lower socioeconomic backgrounds. However, we did not find significant differences in completion rates across families with high or low income in our sample. Individuals who do not have access to devices they can use to respond to surveys or who do not live in an area with sufficient internet coverage might not be represented in similar studies. Further research is therefore needed to determine response rates in more representative samples.

We also found that completion rates were higher on weekdays compared to weekends, though completion rates remained high on weekends. Our results are consistent with previous EMA studies in adults that have reported a similar pattern [42] but differ from a previous study of mothers and children, which found that child compliance with EMA prompts was higher on weekends than on weekdays [43]. Given that differences in affect and activity across weekends and weekdays have also been reported in EMA studies in children and adults [42,44], researchers may consider weekday versus weekend as a control variable in their analyses.

In terms of survey delivery methods, approximately half of the sample preferred to receive survey links by email, whereas the other half preferred to receive notifications by SMS text message. Of note, completion rates were significantly higher for participants receiving SMS text message survey notifications compared to email notifications. Moreover, participants took less time to respond to survey notifications received by SMS text message than by email. Most existing EMA studies have not reported completion rates by notification method (email vs SMS text message), have not provided participants with different notification options [16,45,46], or have used mobile apps with built-in prompts for participants to take assessments at appropriate intervals [47,48]. However, our results are consistent with 1 previous study on drug use among adults in nonurban areas, in which EMA completion rates were higher when notifications were sent through SMS text message compared to email [49]. Therefore, while there is a small cost associated with using Twilio, the add-on program that can be used in conjunction with REDCap to send survey notifications through SMS text message, providing this option may be important for increasing participant responses.

We used 2 survey reminders with the goal of increasing participant response rates, recognizing that participants may not have been available to answer questions immediately at the designated survey times (7:30 AM and 7:30 PM). The first reminder resulted in a substantial increase in responses, whereas the increase in responses following the second reminder (1 hour after the initial survey notification) was more modest. However, the survey also remained active for 3 hours after the initial notification, and we observed that a substantial proportion of participants responded between 2 and 3 hours after the initial survey notification. As a result, having a slightly extended response window may enhance completion rates, though potential effects on participant recall accuracy should be considered.

Lastly, we found that the number of assessments completed was higher in dyads with girls than in dyads with boys. Our results are consistent with meta-analytic evidence that compliance rates are higher in samples containing more women than men [38]; however, our results differ from a previous study that found no difference in compliance with EMA prompts for children or mothers by child gender [43]. The reasons for the gender differences in response rates in our sample are unclear, and should they be replicated in other studies, further research will be needed to understand the reasons for any gender differences in response rates [38]. In terms of parent gender, most parents in our sample were mothers, consistent with the focus of the CHILD Study on perinatal enrollment and data collection. Fathers have been historically underrepresented in research on children’s mental health [50], and further research is needed to understand fathers’ response patterns to EMA surveys.

### Limitations

A limitation of the design we present is that it does not recognize the time zone in which participants are located, which required us to add a separate arm for each time zone. This limitation creates downstream challenges. For example, data cleaning is required to remove redundant, empty fields from other time zones. Additionally, another problem can arise if a participant enters the study on a specific date (say, July 5), and it is already the next day in the time zone of the institution (say, July 6). REDCap will assume that the participants skipped day 1 and will send them the day 2 survey. As a result, daily monitoring of the REDCap project platform by a project administrator is required to manually adjust for this known issue. Finally, participants who travel from one time zone to another might not inform researchers of their move, which compromises the timing at which surveys are completed. If researchers become aware of a time zone change, they will have to manually change the timing of surveys as REDCap queues the surveys daily. Therefore, a limitation of this REDCap design is a lack of full automation, which requires some manual upkeep to ensure that the project runs smoothly and data are captured accurately. It is important to note that newer versions of REDCap support a feature called MyCap, which can be a valuable tool for EMA studies that can facilitate a more efficient and less labor-intensive design. Participants can download the MyCap app on their mobile devices and complete surveys through it. At the time of writing, our institution had not yet supported MyCap. Since versions of REDCap differ from one institution to another, researchers can connect with their REDCap administrators to learn about the capabilities of the REDCap version their institution has.

Aside from design limitations, it is important to note that our sample of participants lacked socioeconomic diversity. While a previous EMA study involving a racially diverse sample found no differences in survey compliance based on participant demographics or other characteristics [51], there remains a dearth of research on the reliability of EMA data collection across diverse samples. Finally, we did not collect information on why participants missed some of the EMA surveys. This information could be important for improving survey completion rates. Future studies can use a feedback survey at the end of the study to inquire why participants were not able to complete all surveys or whether they have recommendations that can facilitate higher completion rates.

### Conclusions

REDCap can be used by researchers for EMA and is flexible enough to accommodate multiple time zones and survey delivery methods (email and SMS text message). The use of SMS text message survey notifications appears to be an important way to increase participant responses. REDCap is freely available to nonprofit organizations that join the REDCap consortium, and there is only a small cost associated with the use of Twilio for SMS text message survey delivery. Moreover, its data storage and administrator controls reduce potential privacy concerns associated with EMA. Researchers wishing to implement an EMA in REDCap should consider the capabilities of this platform and of the REDCap version to which they have access, and the extent to which these meet the requirements of their project design.

## Appendix

Multimedia Appendix 1 Instructions for ecological momentary assessment set-up in REDCap.

## Abbreviations

CHILD: Canadian Health Infant Longitudinal Development
EMA: ecological momentary assessment
REDCap: Research Electronic Data Capture

## Data Availability

The data generated and analyzed during this study are not publicly available because this is an early data report on a subsample from an ongoing study. The full data set will be made available by the corresponding author once the study is completed.
