# Supplementary material for: Feasibility of Using Research Electronic Data Capture (REDCap) to Collect Daily Experiences of Parent-Child Dyads: Ecological Momentary Assessment Study
Source: JMIR Form Res. 2023 Nov 9;7:e42916. doi: 10.2196/42916 (PMC10667976; doi:10.2196/42916)
Supplement: Multimedia Appendix 1 [file formative_v7i1e42916_app1.docx]

**Multimedia Appendix:**

***Ecological Momentary Assessment with REDCap: Methods, Feasibility, and User Behaviour in a Parent and Child Study***

The steps below can be used to set up an ecological momentary assessment (EMA) project in REDCap. These instructions are based on REDCap Version 11.1.21.

**1. Start by creating data instruments in the *Online Designer* tab.**

**
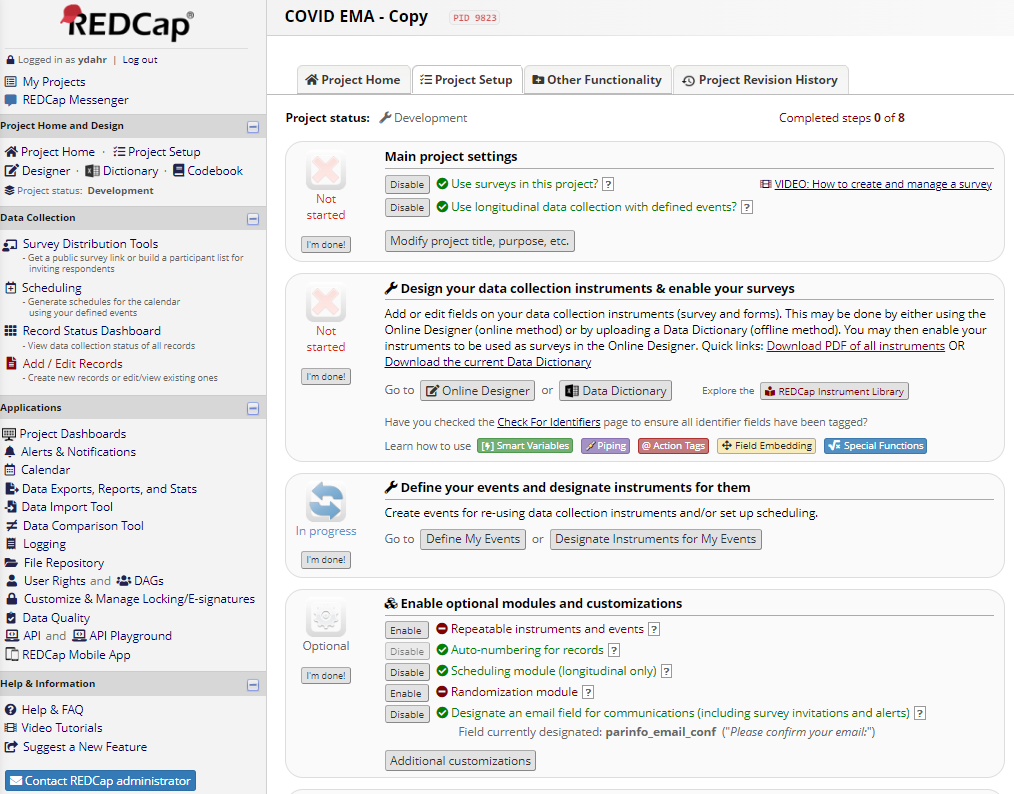
**

Below, you will see our list of instruments. The *Initial Questionnaire* and the *Parent Information and Contact Preferences* form are the first two we set up. We also set up a unique *Morning* and *Evening Survey* for each of the time zones.

**
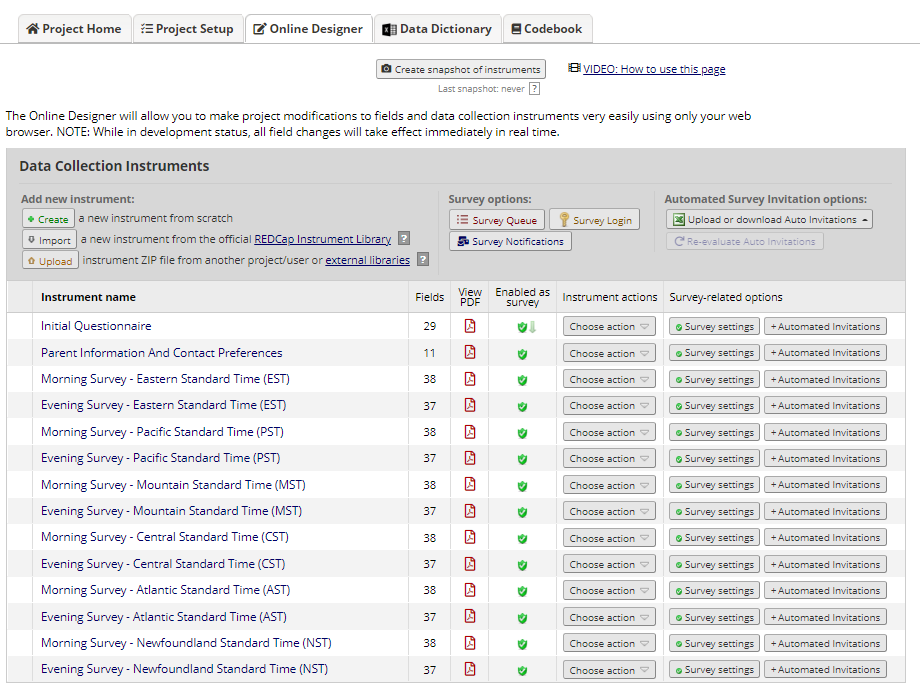
**

**Auto-Populate Date of Completion.** The *Initial Questionnaire* asks participants for their demographic information. After the Record ID field (which is the default first field in the first instrument of a REDCap project), we defined the Date field as follows:


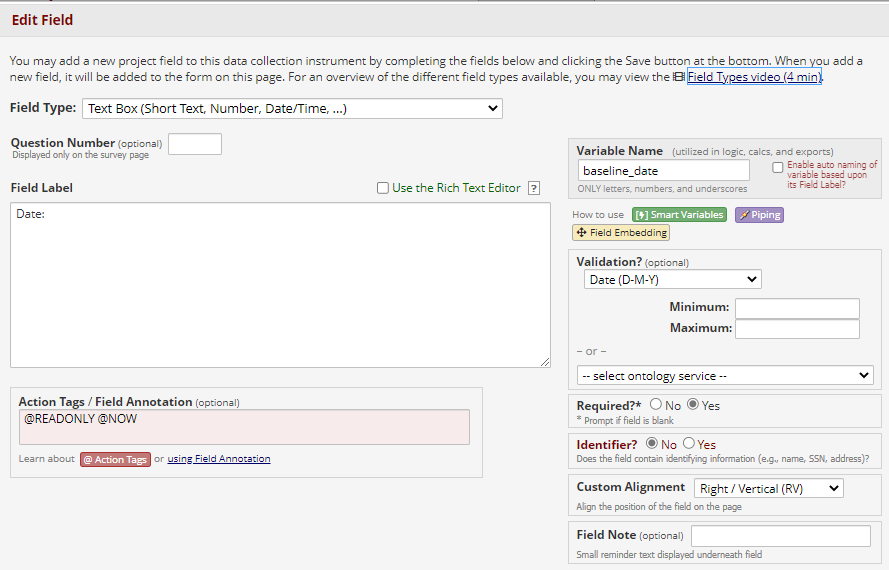


We input the @READONLY and @NOW actions tags so that the date is auto-filled for participants when they complete this baseline survey. This date field will be included in the Automated Survey Invitation logic later on, to define for REDCap how to time the daily surveys.

**Collect Contact Information.** The *Parent Information and Contact Preferences* form allows participants to input their contact information and define their preference for survey delivery method (email or text message). We recommend including “confirm your email/cellphone number” fields to ensure that participants are inputting the correct contact information. Survey delivery setup in later steps will be based on these two confirmation fields.

**
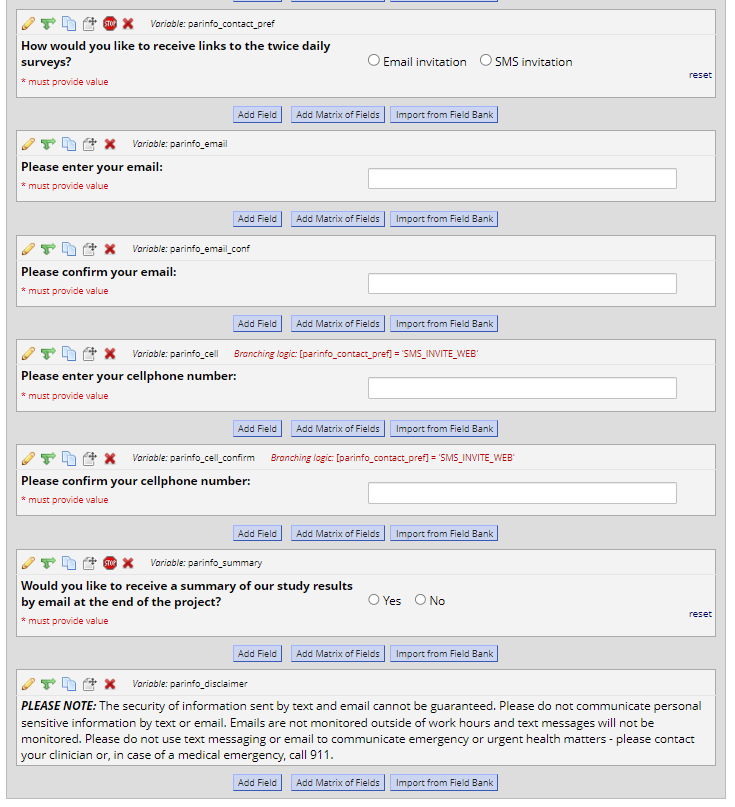
**

**2. Project-level and Survey-level Settings to Consider**

At the top of the Project Setup tab, you will find the options below. Enable both “Use surveys in this project” and “Use longitudinal data collection with defined events.”

**
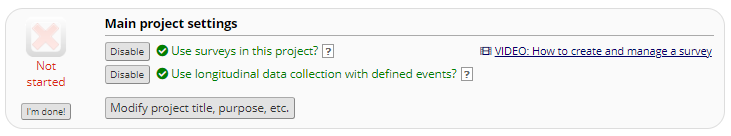
**

Once surveys are enabled, you can go back to the Online Designer page to enable all the data instruments as surveys. Since our study required that participants enter their own data from start to finish, all the instruments were enabled as surveys. You will find the option to enable surveys in the “Enabled as survey” column shown below:


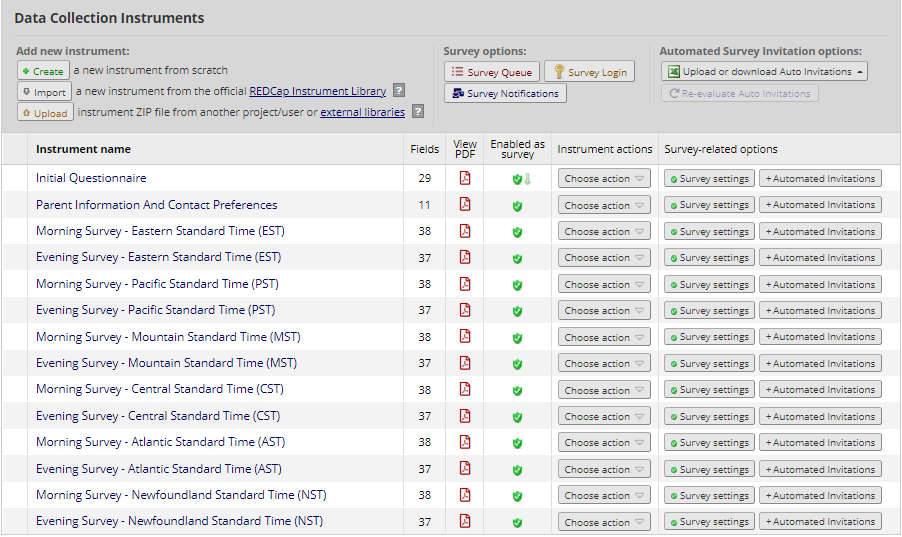


**Set Time Limit for Completion.** Once surveys are enabled, access the “Survey settings” tab in the second-last column on the right-hand side for each of the *Morning* and *Evening Surveys*. In the “Survey Access” portion of these settings, there is an option to limit the time that participants have to complete the surveys after receiving them. We set this limit to three hours:


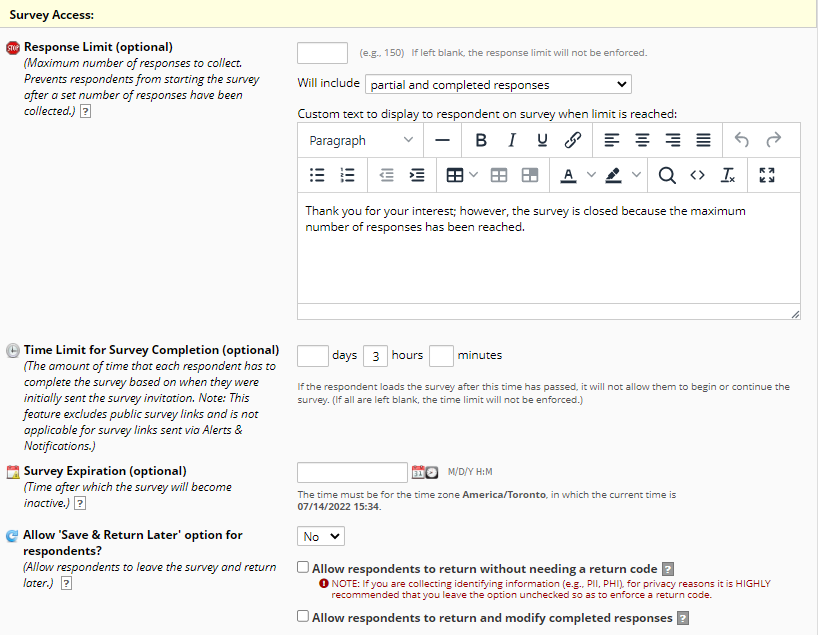


**Add Study-Specific IDs If Needed.** Back on the Project Setup page, in the “Enable optional modules and customizations” section, you will notice that REDCap has enabled “Auto-numbering for records.” This is because we enabled the first data instrument as a survey. If the project requires study-specific IDs in REDCap, these will need to be added to each record manually once the project is in production and data collection is ongoing.


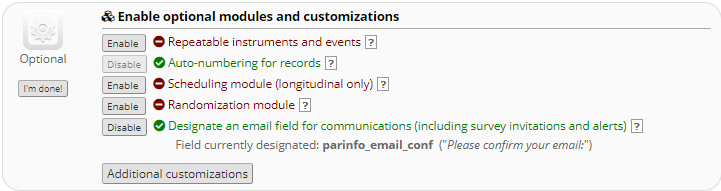


**3. Set Up Survey Notification Delivery by Email or Text Message**

**Text Message Notifications with Twilio.** Depending on your institution’s REDCap settings, Twilio may first need to be enabled in your REDCap project by a REDCap administrator. Once enabled, the Twilio settings section will appear on the Project Setup page.

Click on “Configure Twilio settings” to begin customizing Twilio for your project.


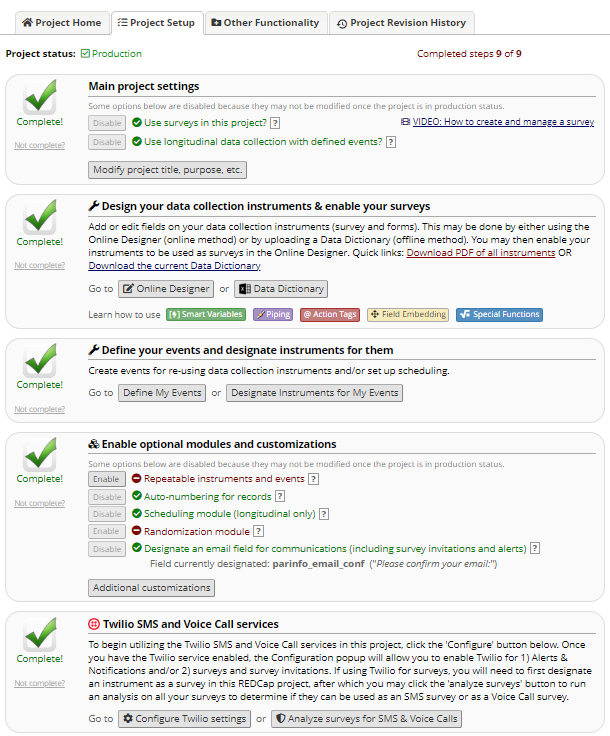


The window below will pop up. The settings our project required were as follows: 1) Twilio enabled for Surveys and Survey Invitations; and 2) Sending the survey link as a link to a webpage.


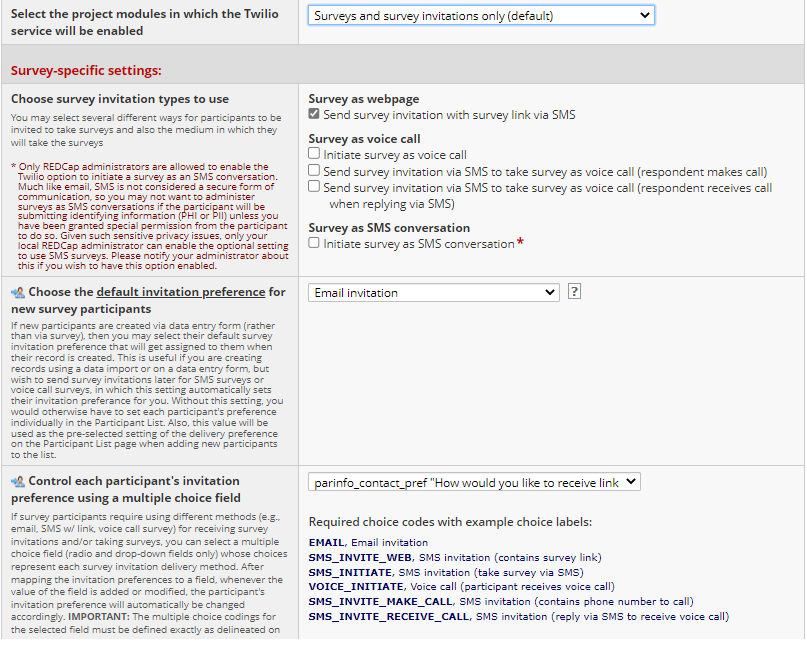

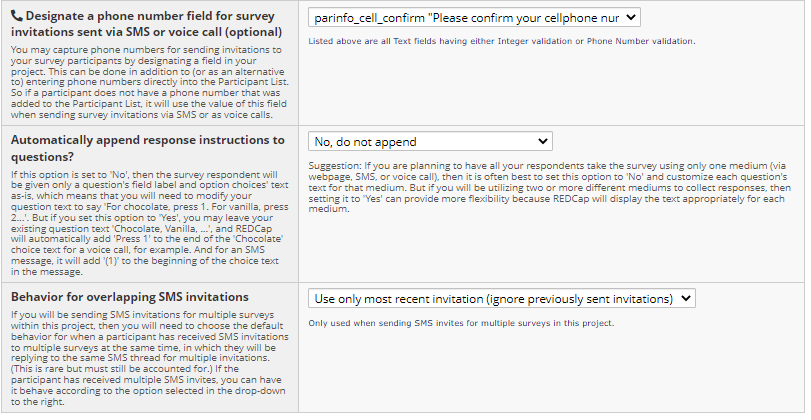


**Identifying Contact Method.** The section in the screenshot below labeled “Control each participant’s invitation preference using a multiple choice field” is used to identify whether each participant receives survey notifications by email or text message. Choose the field from your instruments that participants use to identify their preference.


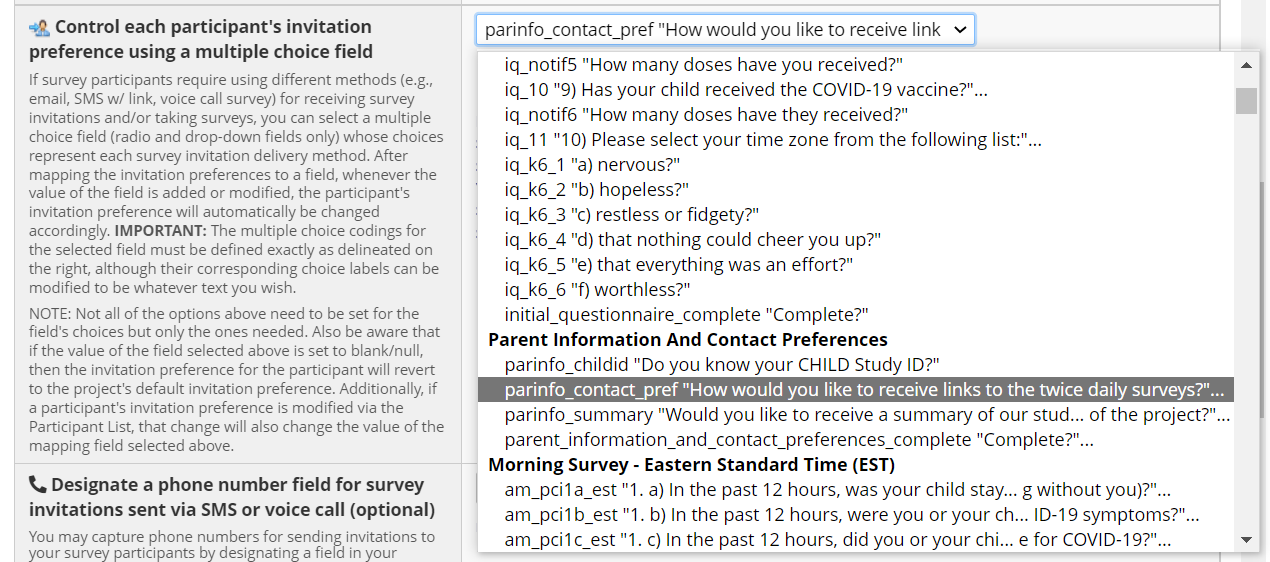


Designate a phone number field that Twilio can use to deliver surveys by text (this field is the cellphone number confirmation field in our *Parent Information and Contact Preferences* form).


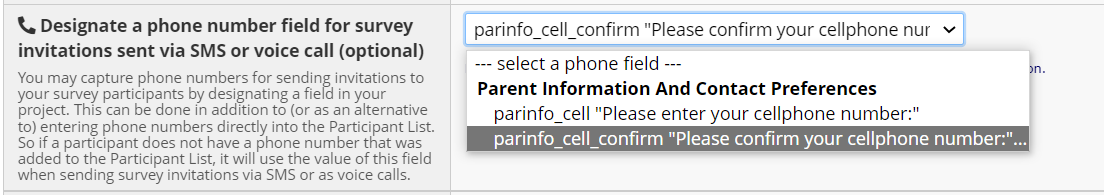


**Email.** Under the “Enable optional modules and customizations” survey settings section on the Project Setup page, enable the “Designate an email field for communication” feature.


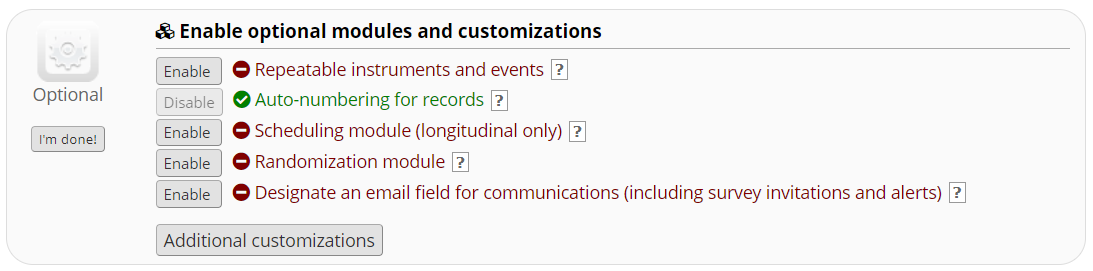


In the window that pops up (shown below), we defined for REDCap the email confirmation field, found in the *Parent Information and Contact Preferences* form that was mentioned in Step 1, that would be used for email delivery of surveys.


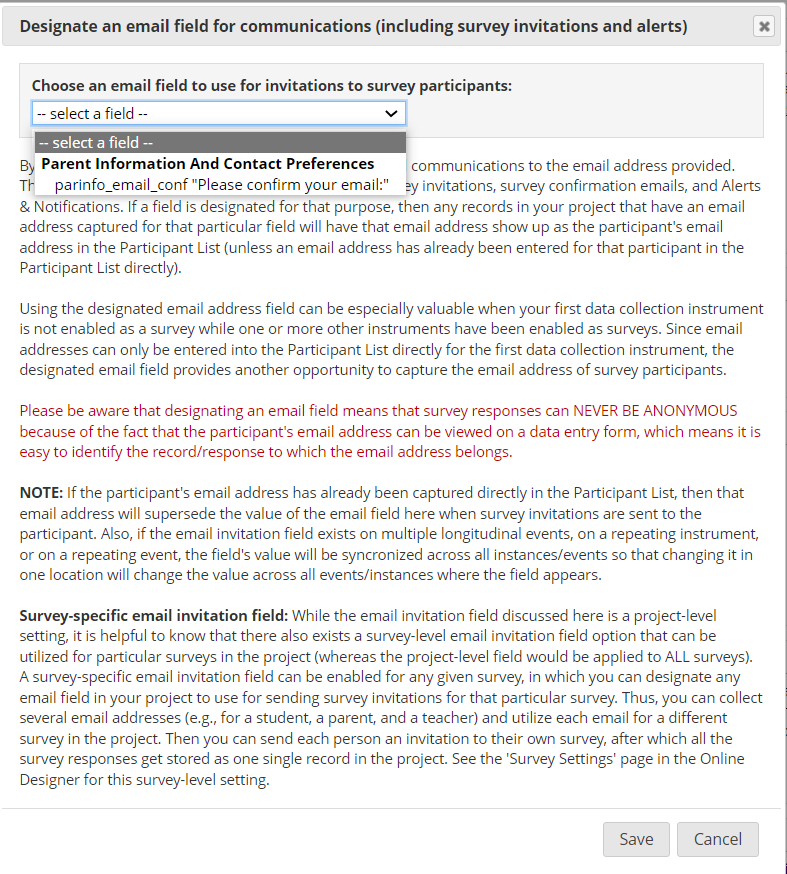


**4. Set up Events and Designate Instruments for Them**

This step is used to identify which surveys will be completed at each time point. On the Project Setup tab, you’ll find the “Define your events and designate instruments for them” options. Click on “Define my events.”


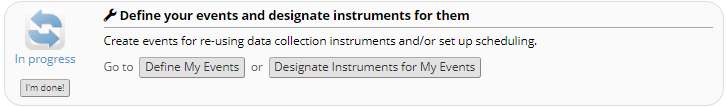


**Setting Up Surveys across More Than One Time Zone.** If your project will be completed across multiple time zones, you will first need to set up separate arms (one for each time zone). Use the “Add new arm” function to create an arm for each time zone. If your project will be completed in a single time zone, additional arms are not required.


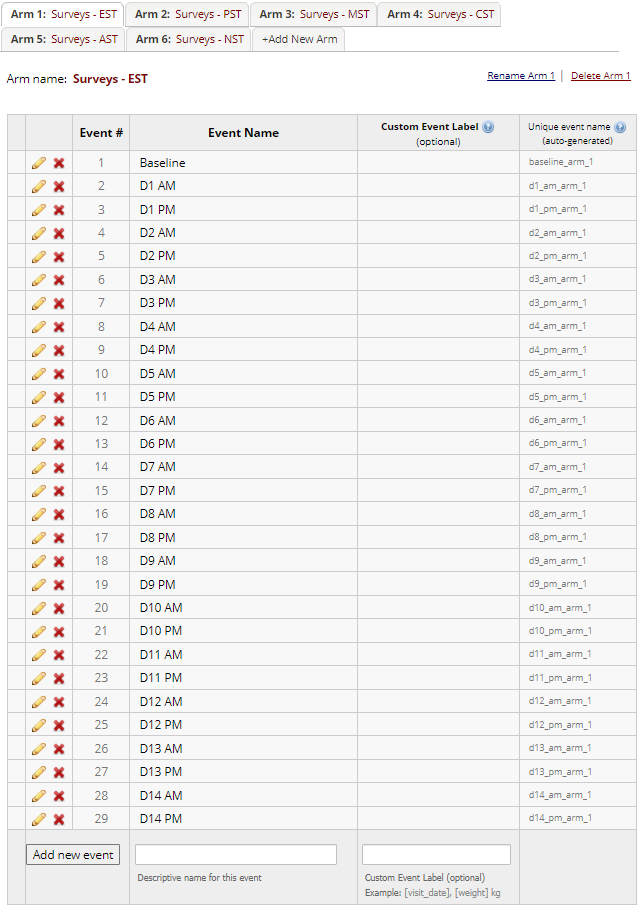


**Defining Events for Each Arm.** Beginning in the first arm (if multiple arms have been set up to accommodate multiple time zones), use the “Add new event” button at the bottom to add one event per assessment.

The total number of events will be equal to the number of days participants are in the study multiplied by the number of assessments per day, plus one (for the baseline survey, in which participants provide contact preferences and, if required, additional background information). In our case, we had 29 events (14 days X 2 assessments + 1 baseline survey).

Note the unique event name that REDCap generates each time an event is created. These are important for setting up the logic in the Automated Survey Invitations in the next step.

**Identify Instruments for Each Event.** Once all the events are set up in each of the arms, go to the “Designate instruments for my events” tab at the top of the page.


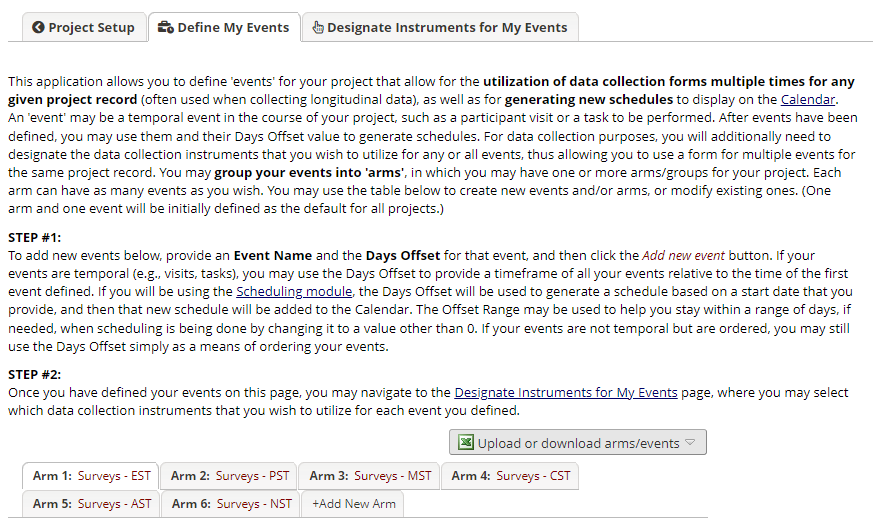


Under this tab, you can assign the instruments to the appropriate event in each arm. The instruments you will see listed are the exact ones created in the Online Designer.

In the example below, the EST time zone arm is shown. For this arm, we assigned the *Initial Questionnaire* and *Parent Information and Contact Preferences* form to the Baseline event, then we assigned the *Morning Survey – EST* and *Evening Survey – EST* to their respective events, by checking the appropriate boxes underneath each event. Repeat this for each of the arms.


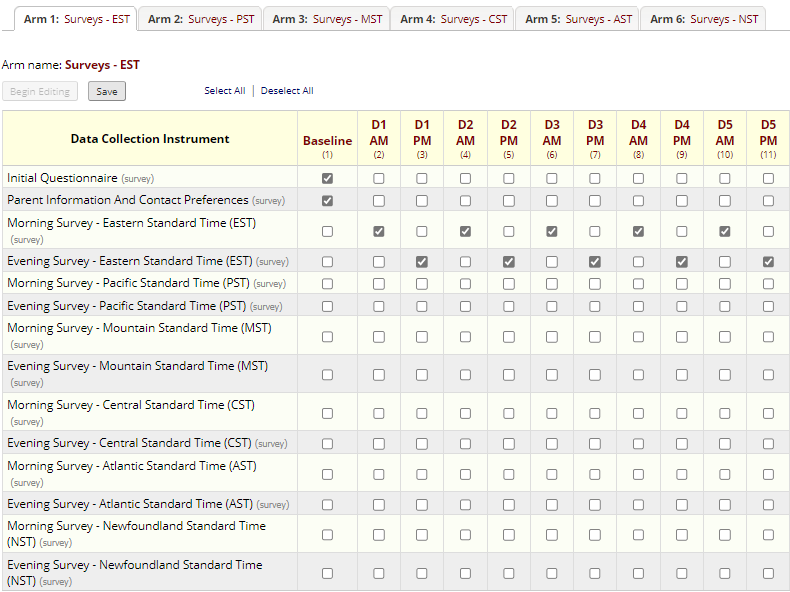


Note that, since our study had to accommodate multiple time zones and two different daily surveys, this project had 12 instruments set up in total (aside from the Baseline instruments). Studies occurring in one time zone alone and/or requiring only one recurring survey will not have this many instruments listed under the “Designate instruments for my events” tab.

**5. Program the Automated Survey Invitation for each Event**

Returning to the Online Designer page, each data instrument has corresponding Automated Invitations that can be programmed for each of the events that were defined in the previous step. We will begin by programming the invitations for the Day 1 *Morning Survey* in the EST time zone:


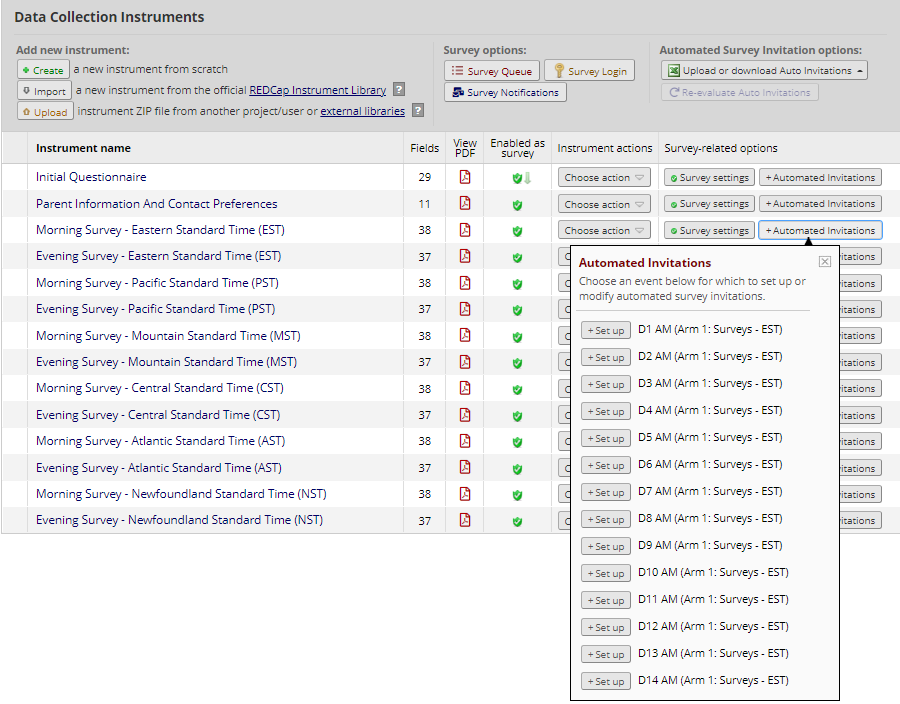


The window that pops up will appear as such:


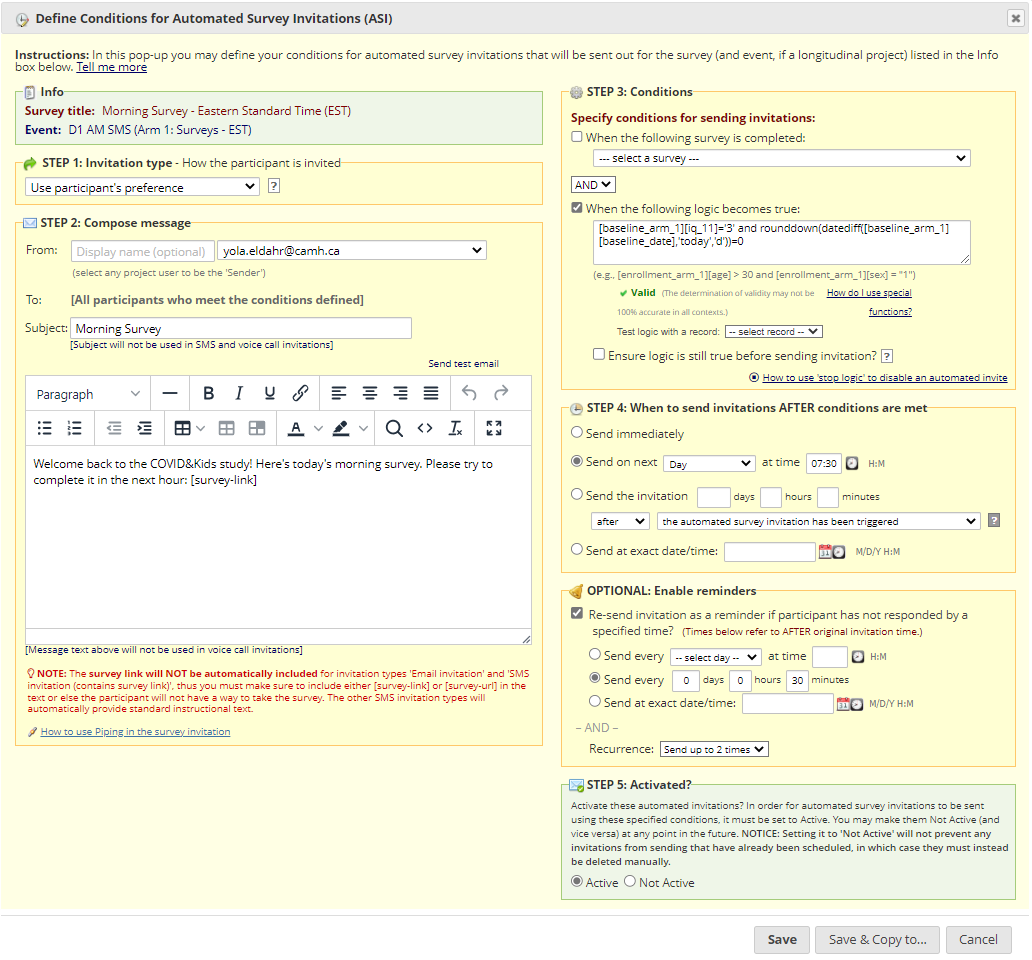


The settings should be as follows:

- **Step 1: Invitation type**

Choose “Use participant’s preference” so that the project accommodates both Email and SMS survey delivery.

- **Step 2: Compose message**

Enter the message you want participants to receive via email or text message.

- **Step 3: Conditions**

Click on “When the following logic becomes true” and modify our example logic to identify when automated survey invitations will be sent:

- [baseline_arm_1][iq_11]='3': This variable represents question 11 in our *Initial Questionnaire* [iq_11], which asks participants to confirm their time zone. This variable resides in the Baseline event of Arm 1 (EST time zone) [baseline_arm_1].
- rounddown(datediff([baseline_arm_1][baseline_date],'today','d'))=0: this formula triggers REDCap to calculate the difference between today’s date and the date that the *Initial Questionnaire* was completed. A difference of 0 triggers the Day 1 surveys to be cued. A difference of 1 triggers the Day 2 surveys to be cued, and so on.

*Note: There is one exception where this formula will not work. If a participant completes their Baseline event in a different time zone at a time before midnight, but the timing in the institution’s time zone is after midnight, REDCap will calculate a difference of 1, then it will queue the Day 2 surveys to be sent and skip over Day 1. In this instance, Day 1 surveys must be set up for delivery manually, for each record where this is the case.

- **Step 4: When to send invitations AFTER conditions are met**

Define for REDCap at what time the morning survey will be sent

- In our project, the initial morning survey notifications were sent at 7:30 AM each day.
- REDCap time and date settings are based on those of the institution for which it is set up; therefore, notification timings for the other time zones must account for time differences. For example, to send surveys at 7:30 AM MST, the notification time must be set to 9:30 EST because our institution is in the EST time zone, and the MST time zone is two hours behind us.
- **OPTIONAL: Enable reminders**

Check “Re-send invitation as a reminder if participant has not responded by a specified time.” We enabled up to two reminders, to be sent to participants at 30-minute intervals from the time of initial survey delivery. These notifications are only sent if participants have not responded to the previous notification that was sent.

- **Step 5: Activated?**

Select “Active” to enable the notifications.

Repeat these steps for each of the events listed in the Automated Invitations drop-down menu of each instrument.

Note that you can save time on the setup of subsequent events’ Automated Invitations. After setting up the first survey invitation in a series corresponding to each instrument, you can use the “Save and Copy to” option in the bottom right of the settings window to paste the settings for the rest of the events.


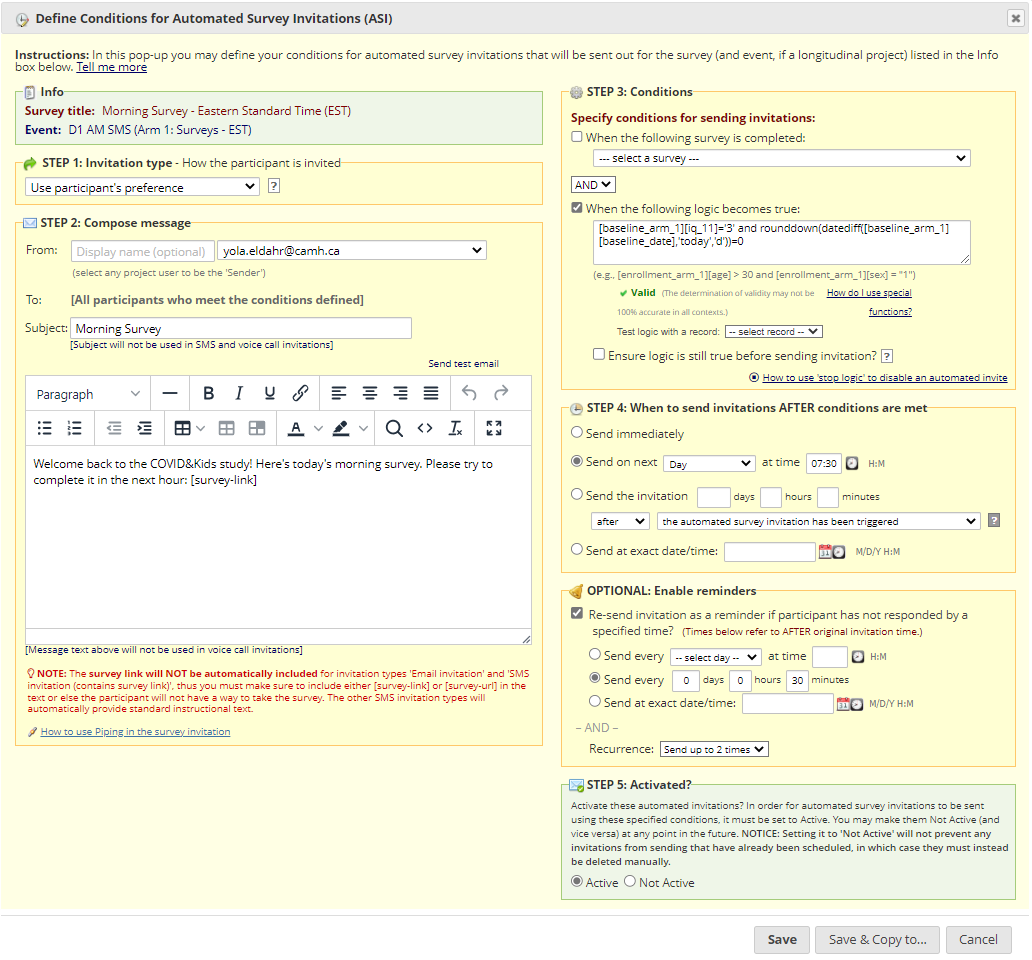


The only change you will have to make for each survey invitation is in the “When the following logic becomes true” text box. You will have to change the number at the end of the rounddown variable to correspond with the day the survey is supposed to be sent.

**6. Thoroughly pilot your project setup**

We recommend that you test out your REDCap project extensively once setup is done, for all time zones your project may include and for both Email and SMS delivery.
